# Relief of cardiac tamponade by a congenital partial left-sided pericardial defect in a patient with ruptured acute type A aortic dissection: a case report

**DOI:** 10.1186/s40981-019-0223-4

**Published:** 2019-01-11

**Authors:** Kota Nishimoto, Takeshi Umegaki, Sayaka Ohira, Yurina Nakajima, Takehiro Soeda, Takahiko Kamibayashi

**Affiliations:** Department of Anesthesiology, Kansai Medical University Hospital, 2-3-1 Shin-machi, Hirakata, Osaka 573-1191 Japan

**Keywords:** Congenital pericardial defect, Acute type A aortic dissection, Cardiac tamponade

## Abstract

**Background:**

Acute type A aortic dissections have an extremely poor prognosis, and cardiac tamponade is a major cause of death in these patients. Here, we describe a case where congenital partial pericardial defect relieved cardiac tamponade caused by ruptured type A aortic dissection.

**Case presentation:**

A 79-year-old woman was hospitalized after experiencing chest pains and respiratory distress. She developed out-of-hospital cardiopulmonary arrest and was resuscitated with no sequelae 5 days before admission. Computed tomography confirmed pericardial and left pleural effusions, and type A aortic dissection was diagnosed. We began emergency ascending aortic replacement surgery under general anesthesia with propofol and remifentanil and incidentally discovered a congenital partial left-sided pericardial defect that allowed drainage of the hemopericardium and relieved cardiac tamponade. The surgery was successfully performed, and the patient recovered without complications.

**Conclusions:**

We experienced an extremely rare case where a congenital partial pericardial defect relieved cardiac tamponade associated with aortic dissection and contributed to the patient’s survival.

## Background

Acute type A aortic dissections are associated with an extremely poor prognosis, and the mortality rate increases 1–2% per hour after symptom onset [1]. Cardiac tamponade is a major cause of death in the acute phase [1]. Here, we report a case of type A aortic dissection accompanied with pericardial and left pleural effusion. During emergency surgery, we discovered a congenital partial left-sided pericardial defect, which would lead to spontaneous drainage of cardiac tamponade.

## Case presentation

The patient was a 79-year-old woman with a height of 153 cm, weight of 50.0 kg, and body mass index of 21.4 kg/m^2^. She had previously undergone surgeries for cerebral meningioma and cervical spondylosis. One year earlier, the patient had experienced a transient ischemic attack accompanied by transient amaurosis and was prescribed aspirin. A transthoracic echocardiogram at that time found no wall motion abnormalities, an ejection fraction of 67%, and mild-to-moderate aortic regurgitation.

Five days before admission to our hospital, the patient experienced an out-of-hospital cardiopulmonary arrest. A family member performed cardiopulmonary resuscitation, and the patient regained consciousness. The patient denied further medical treatment at that time. However, due to continued chest pains and progressively worsening respiratory distress, the patient visited our institution. Her vital signs on arrival were as follows: temperature of 36.0 °C, pulse of 97 bpm, blood pressure of 159/88 mmHg, and respiratory rate of 26/min.

Blood analysis showed a reduction in hemoglobin level (7.9 g/dL; normal range 12.5–15.5 g/dL), elevated levels of plasma cardiac troponin I (52 pg/mL; normal range 0–26 pg/mL), and N-terminal prohormone of brain natriuretic peptide (5600 pg/mL; normal range 0–125 pg/mL). Arterial blood gas analysis (room air) showed PaO_2_ of 65.0 mmHg and PaCO_2_ of 47.6 mmHg on arrival at the hospital. A transthoracic echocardiogram indicated pericardial effusion, but showed no other new developments since the previous echocardiogram.

**Fig. 1:**
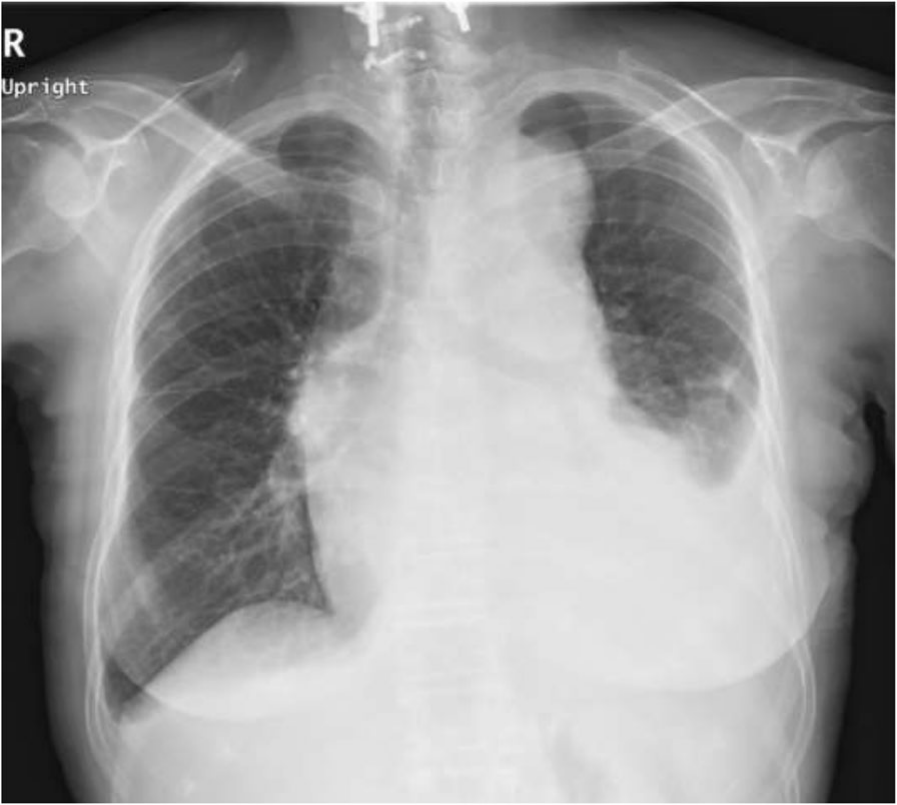
Preoperative chest X-ray showing blunting of the left costophrenic angle

**Fig. 2:**
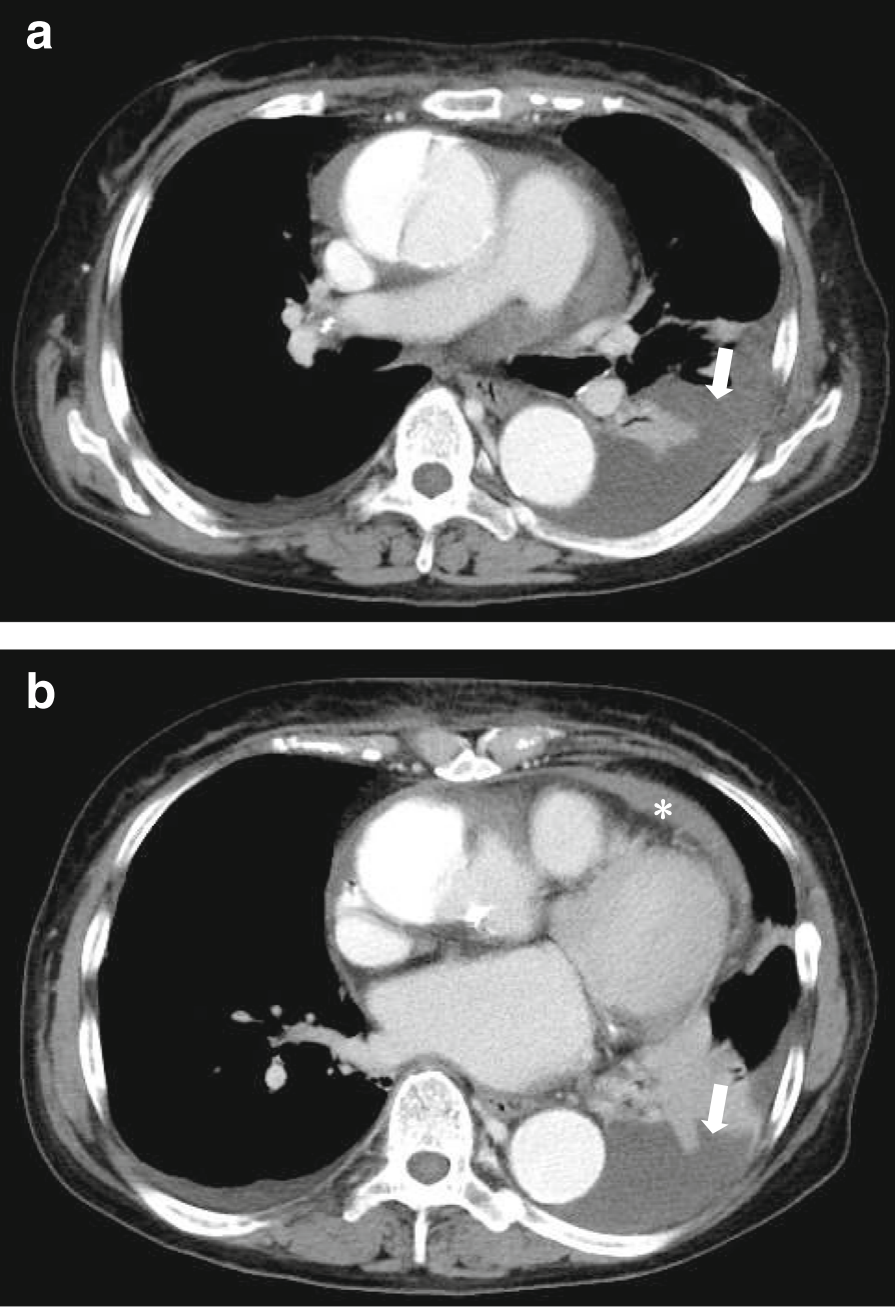
Enhanced computed tomography scans demonstrating aortic dissection and pleural effusion (arrow) (**a**) and pericardial effusion (asterisk) (**b**)

A chest X-ray showed blunting of the left costophrenic angle (Fig. 1), indicating pleural effusion. Contrast-enhanced computed tomography (CT) revealed type A aortic dissection with a dilated ascending aorta (55 mm) accompanied with pericardial and left pleural effusion (Fig. 2a, b shows CT scans of the chest taken at different points). The pericardial and pleural effusions showed no enhancement. The diameter of the ascending aorta was 55 mm. We strongly suspected that the out-of-hospital cardiopulmonary arrest experienced by the patient had been the result of pericardial effusion culminating in cardiac tamponade. Following the diagnosis of acute type A aortic dissection, the patient was immediately prepared for ascending aortic replacement surgery.

In the operating room, the patient was hemodynamically stable during induction of general anesthesia with midazolam (3 mg), remifentanil (0.3 μg/kg/min), and rocuronium (40 mg). Anesthesia was then maintained with sevoflurane (1.5–2.0%) and remifentanil (0.1–0.3 μg/kg/min). We used propofol (2–4 mg/kg/h) instead of sevoflurane during cardiopulmonary bypass. Transesophageal echocardiography demonstrated slight pericardial effusion and a distinct echo-free space that indicated left pleural effusion.

Surgery was initiated via median sternotomy with the patient in supine position. After the initial incision, a dark red-colored hematoma (approximately 45 g) was observed in the pericardium, and a larger hematoma (approximately 600 g) was found in the left pleural cavity. We then discovered a 20 × 15-mm defect in the pericardium toward the left pleural cavity and diagnosed a congenital partial left-sided pericardial defect. A blood clot had formed around the defect that appeared to be obstructing further blood flow. A 15-mm tear was found adjacent to the ascending aorta, and ascending aortic replacement surgery was performed under cardiac arrest using hypothermic cardiopulmonary bypass (20 °C). As the left ventricle and left atrial appendage were seemingly unaffected by the pericardial defect, pericardial reconstruction was not carried out. The duration of surgery, anesthesia, and cardiopulmonary bypass was 376, 497, and 254 min, respectively.

After surgery, the patient was transferred to the general intensive care unit and was weaned from mechanical ventilation on the third postoperative day. CT scans after 7 days showed that the pleural effusion had resolved completely, and an echocardiogram after 14 days did not detect any pericardial effusion. The patient recovered without any complications and was discharged 26 days after surgery.

## Discussion

Several studies have previously reported ruptured acute aortic dissection cases associated with a partial or complete congenital pericardial defect [2–6]. However, those cases involved little or no pericardial effusion with massive left hemothorax. In contrast, our patient presented with substantial pericardial effusion, and the hemothorax could not be described as “massive.” The pericardial defect appeared to have relieved cardiac tamponade by allowing drainage of the hemopericardium into the pleural space. The hemorrhage from the damaged section of the aorta had likely spontaneously resolved, leading to the formation of a blood clot that prevented further bleeding. We posit that this prevented the patient from going into hemorrhagic shock during the hyperacute phase of aortic dissection, thereby contributing to her survival.

Congenital absence of the left pericardium results from the incomplete formation of the pleuropericardial membrane, which is caused by premature atrophy of the left duct of Moore [7]. Congenital pericardial defects occur in fewer than one in 10,000 cases [8] and present three times more frequently in men than in women [9]. Complete left-sided pericardial defects are the most common, accounting for 70% of all reported defects [10]. However, many asymptomatic cases of congenital pericardial defects escape detection, and a large number of these may involve partial left-sided defects that are small and difficult to diagnose even if they present as minor electrocardiogram abnormalities [11].

Asymptomatic pericardial defects, whether partial or complete, generally have good prognoses, and many surgeons therefore choose to continue monitoring these patients without surgical intervention. Surgical treatment is, however, indicated for symptomatic cases, where pericardiotomy, pericardial reconstruction, or pericardial closure is performed to prevent compression of the coronary blood vessels and herniation of the left atrium and ventricle. Studies have reported phrenic nerve paralysis due to phrenic nerve compression associated with a partial left-sided pericardial defect as well as a cleft mitral valve associated with a right-sided pericardial defect [12, 13]. Previous studies have also reported the incidental discovery of asymptomatic pericardial defects in pneumothorax cases or during thoracic and cardiovascular surgery [14–16]. The discovery of the partial left-sided pericardial defect in our patient was also an incidental surgical finding.

## Conclusions

We reported an extremely rare case where a congenital partial left-sided pericardial defect relieved cardiac tamponade associated with acute type A aortic dissection and allowed the patient to avoid hemorrhagic shock during the hyperacute phase. Life-saving ascending aortic replacement surgery was successfully performed immediately after diagnosis.

## Abbreviations

CT: Computed tomography
PaCO_2_: Partial pressure of arterial carbon dioxide
PaO_2_: Partial pressure of arterial oxygen

## References

[1] Hagan PG, Nienaber CA, Isselbacher EM, Bruckman D, Karavite DJ, Russman PL (2000). The International Registry of Acute Aortic Dissection (IRAD): new insights into an old disease. JAMA.

[2] Nakajima M, Tsuchiya K, Naito Y, Inoue H, Kobayashi K, Mizutani E (2004). Partial pericardial defect associated with ruptured aortic dissection of the ascending aorta: a rare feature presenting severe left hemothorax without cardiac tamponade. Ann Thorac Surg.

[3] Matsuda N, Marumoto A, Nakashima H, Nakamura Y, Kamihira S, Ishiguro S (2004). Congenital pericardial defect associated with ruptured type A dissection. Ann Thorac Surg.

[4] Nisanoglu V, Erdil N, Battaloglu B (2005). Complete left-sided absent of the pericardium in association with ruptured type A aortic dissection complicated by severe left hemothorax. Tex Heart Inst J.

[5] Huang JW, Chang PC, Chiu CC, Wang PH, Hsieh CC, Chen HM (2007). A rare anatomical entity of congenital pericardial defect with acute type A aortic dissection. Chirurg.

[6] Furui M, Ohashi T, Hirai Y, Kageyama S (2012). Congenital pericardial defect with ruptured acute type A aortic dissection. Interact Cardiovasc Thorac Surg.

[7] Moore RL (1925). Congenital deficiency of the pericardium. Arch Surg.

[8] Yamano T, Sawada T, Sakamoto K, Nakamura T, Azuma A, Nakagawa M (2004). Magnetic resonance imaging differentiated partial from complete absence of the left pericardium in a case of leftward displacement of the heart. Circ J.

[9] Sunderland S, Wright-Smith RJ (1944). Congenital pericardial defects. Br Heart J.

[10] Nasser WK. Congenital diseases of the pericardium. Cardiovasc Clin. 1976;7:271–86. 826317

[11] Tubbs OS, Yacoub MH (1968). Congenital pericardial defects. Thorax.

[12] Ogawa J, Nagashima H, Suhara M, Yamauchi K (2009). A case of left phrenic nerve paralysis caused by compression of the left auricle due to congenital partial left pericardial defect. Nihon Kokyuki Gakkai Zasshi.

[13] Kaimoto S, Kawasaki T, Yamano M, Miki S, Kamitani T, Sugihara H (2013). A case of congenital absence of the right pericardium with mitral valve cleft. J Cardiol Jpn Ed.

[14] Murasawa M, Yoshizawa M, Ishida H, Kuwabara M (2016). Congenital defect of the left pericardium with spontaneous pneumothorax; report of a case. Kyobu Geka..

[15] Furugen T, Teruya T, Hirayasu T, Yamashiro S, Kuniyoshi Y (2011). Congenital partial defect of the left pericardium with spontaneous pneumothorax; a case report. J Jpn Surg Assoc.

[16] Nakamura K, Uchida T, Mizumoto M, Miyazaki R, Kim C, Yoshimura Y (2013). Pericardial defect incidentally noticed during off-pump coronary artery bypass grafting; report of a case. Kyobu Geka.

